# Preliminary Development of a Patient‐Reported Outcome Measure for Inducible Laryngeal Obstruction: Findings From a Delphi Process

**DOI:** 10.1002/resp.70262

**Published:** 2026-05-19

**Authors:** Siobhan Ludlow, Stephen Fowler, Jamie Kirkham, Lucie Byrne‐Davis

**Affiliations:** ^1^ Manchester University NHS Foundation Trust Manchester UK; ^2^ University of Manchester Manchester UK

**Keywords:** clinical outcome assessments, Delphi methodology, inducible laryngeal obstruction, patient reported outcome measure

## Abstract

**Background and Objective:**

Inducible laryngeal obstruction (ILO) is a laryngeal disorder that intermittently affects breathing. A psychometrically robust patient‐reported outcome measure for ILO is required. We have generated items from individual patient interviews and a scoping review. The objective of this study is to ascertain which items covering the physical, social, and psychological impact of ILO were relevant for measuring the impact of living with ILO to inform a new patient‐reported outcome measure.

**Methods:**

An international two‐round online Delphi study was conducted amongst a group including patients with ILO and relevant health care professionals. Participants judged the relevance of 80 items about the impact of ILO on a 9‐point scale. We defined consensus as a minimum of 70% of participants rating items as 7–9. Participants were asked to give a reason for their ratings.

**Results:**

Forty‐six participants registered for the Delphi survey, with 37/46 (80%) and 29/37 (78%) completing rounds one and two, respectively. Participants were patients living with ILO (63%), respiratory physicians (10%), speech and language therapists (16%), physiotherapists (5%), a psychologist (3%) and a nurse (3%). Consensus was reached after round two for 37/80 (46%) to include, 11/80 (14%) not important to include and 32/80 (40%) being important but not critical to include [therefore 43/80 (54%) excluded].

**Conclusion:**

Stakeholders agreed on 37 items that should be included in a PROM for ILO. Further studies to establish the psychometrics of a PROM based on these items are required.

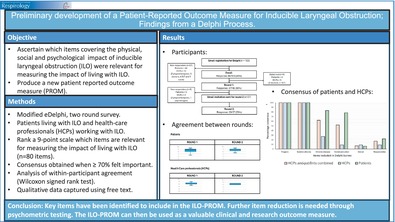

## Graphical abstract

Currently there are few patient‐reported outcome measures for inducible laryngeal obstruction which characterise the multidimensional impact of the disease. The Delphi survey identified which items were important to include from a patient and health care professional perspective. The ILO‐PROM will be a valuable clinical and research outcome measure in the future.

## 1 Introduction

Inducible laryngeal obstruction (ILO) describes a reversible narrowing of the laryngeal aperture in response to external triggers [1]. It results in difficulty breathing, throat tightness, voice disturbance, and chronic cough [2]. Episodes are often sudden [3] and triggered by everyday things such as talking, laughing, exertion, strong scents, temperature changes, or stress [4]. It can also occur without an identified stimulus [5]. These symptoms can impact an individual's ability to exercise, work, socialise, and go out alone [6], along with mood, motivation [7] and overall quality of life [8].

Clinical outcome assessments (COAs) examine the consequences of a disease or disorder in terms of mortality (survivorship) or morbidity (symptom severity, impact of the condition on various health related quality of life and negative consequences of treatment) [9]. It was observed in the literature that there was a wide range of outcome measures used in ILO and a lack of a disease‐specific PROM that captured all domains of the World Health Organisation International Classification of Function, Disability and Health (WHO‐ICF framework).

The most widely used disease‐specific PROM is the Vocal Cord Dysfunction Questionnaire (VCDQ) [3]. This focuses on symptom monitoring in ILO but focuses mostly on the physical impact rather than also including the psychological and social impact. The Exercise‐Induced Laryngeal Obstruction Dyspnoea Index (EILODI) [10] is a PROM developed for patients with exercise‐induced laryngeal obstruction (EILO), but this focuses only on symptoms of ILO related to exercise. The Dyspnoea Index (DI) [11] is a PROM used to measure multi‐dimensional breathlessness. It covers both physical, psychological, and social dimensions but is not disease specific.

**FIGURE 1:**
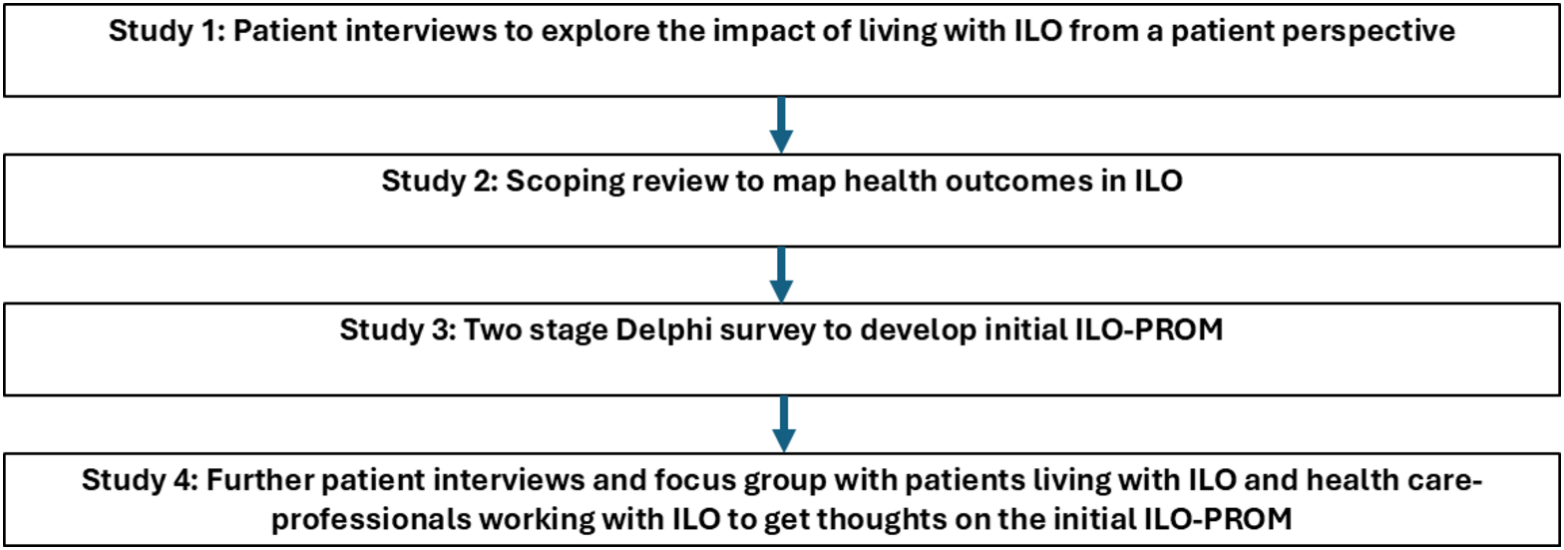
Stages of inducible laryngeal obstruction patient reported outcome measure (ILO‐PROM) development.

A scoping review of existing COAs in ILO demonstrated a need for development of new patient reported outcome measure (PROM) with a specific focus on the biological, individual and social impact [12] in line with the (WHO‐ICF) framework [13]. It was considered important that the development of the new PROM was carried out in collaboration with patients living with ILO and health care professionals (HCPs) working with ILO. The development method consisted of four stages and will then be validated in two further stages (six stages in total) (Figure 1).

We therefore sought to identify, in people with ILO and in HCPs working with the condition, which items measuring the impact of function, disability and health are important and to consider for inclusion in the new PROM.

## 2 Methods

### 2.1 Study Design

A modified electronic‐Delphi (e‐Delphi) process was used to develop the first draft of the Inducible Laryngeal Obstruction Patient Reported Outcome Measure (ILO‐PROM). The Delphi technique was appropriate to address the study aims as it is a method for establishing consensus amongst a group of experts [14].

We used a modified e‐Delphi group design, with the questionnaire administered via a specifically designed Research Electronic Data CApture (REDCap, version 15.5.8, Vanderbilt University) database hosted at Manchester University NHS Foundation Trust. The e‐Delphi approach allowed for global recruitment while preserving the anonymity of the group and ensuring feedback on the surveys remained confidential throughout the Delphi process [14].

Methods and results are reported in adherence with the reporting standard Conducting and Reporting Delphi Studies (CREDES), which promotes consistency and quality in conducting Delphi studies [15].

### 2.2 Study Participants and Recruitment

We aimed to recruit people living with ILO and a range of healthcare professionals working with ILO. A purposive sampling approach was used to elicit diverse perspectives with respect to age group, sex, ethnicity, co‐morbidity, country and health care professional speciality. Patients targeted were adults ≥ 17 years recruited from a regional complex breathlessness service (the Manchester Airways Service, based at Manchester University Foundation Trust, UK). Patients were eligible if they had a diagnosis of ILO confirmed on laryngoscopy. Some patients were recruited at diagnosis [*n* = 10 (43%)], others were completing or had already completed a block of speech and language therapy (SLT), had previously shown an interest in research and were contacted by email to see if they wished to be involved [*n* = 13 (57%)]. Healthcare professionals who were experts in the field of ILO were contacted by email to see if they wished to be involved. An expert was considered someone who worked in a multi‐disciplinary complex breathlessness service with ILO patients. These participants were identified through national and international existing collaborative networks, publicly available hospital and university staff directories and key academic publications in the field of ILO. Thirty‐two health care professionals were contacted by email, most UK based but some of the other international ILO centres were targeted [USA (*n* = 3), Australia (*n* = 2)]. We only contacted UK patients who had attend Manchester University Foundation Trust, as they had previously consented to being involved in research. If interest was expressed by patients or health care professionals, the participant information leaflet (PIL) was sent by email and any questions answered.

We aimed to recruit 20 patients and 10 health care professionals (30 participants) which was established from guidelines on conducting Delphi surveys in health research [14]. Sample size and heterogeneity depend on the purpose of the project, design selected and time frame for data collection [14]. Having patients and a range of different health care professionals achieves a heterogenous sample to ensure the entire spectrum of opinion is determined.

### 2.3 Materials

Eighty items were presented in this Delphi survey. These items were gathered from the findings of a scoping review exploring current health outcome measures used in ILO [12] and a qualitative interview study exploring the impact of living with ILO from a patient perspective [6]. The qualitative interviews highlighted the huge psychosocial burden ILO has on individuals and supported this work focusing on developing an outcome measure to capture the bigger impact of the disease.

The 80 items covering a range of domains of function, disability and health status were first shown to a patient and public involvement and engagement (PPIE) focus group to ensure no items were missing. This consisted of six patients living with ILO who had not been involved in the initial qualitative patient interviews. All items were felt to be relevant, and no items were felt to be missing.

### 2.4 Procedure

An email was sent to 103 individuals (71 patients living with ILO and 32 HCPs with an interest in ILO) who had previously shown at interest in being contacted regarding ILO research opportunities. The e‐Delphi consisted of a two‐round electronic survey. The survey was created in REDCap [16] which is a web‐based tool for secure and flexible data collection. The items were formatted as a 9‐point numerical scale rating how important the factor was in considering the impact of ILO. Scores 1–3 (not important to include), 4–6 (important but not critical), 7–9 (critical to include). There was also the option of ‘unable to score’. Free‐text spaces were provided for participants to expand on their responses. Participants were given two‐weeks to respond between each round with reminder emails sent after one‐week, and 48 and 24 h before closing. Participants who completed or partially completed round 1 were sent the link to the round 2 survey. In round 2 the total number of items remained the same with the participant's previous score, along with the median and interquartile ranges for the health care professionals and patient scores from round 1, provided for each item. Participants were able to change their score if they wished on round 2, and the free‐text option was again provided to expand on reasoning for the score.

### 2.5 Data Analysis

Quantitative data from numerical rating scales were analysed using descriptive statistics generated in Microsoft Excel. The survey was analysed using the Holey and colleagues' method of assessing consensus and stability [17].

1. Percentage response rates.
2. Level of agreement in percentage terms for each item to allow for differing response rates. The percentage of participants scoring the item as ‘critical to include’ (options 7–9) was used to determine the percentage agreement score for each item. Consensus was achieved if an item percentage agreement was ≥ 70%. If patients and health‐care professionals did not agree, that is, patients reached consensus individually but when combined consensus was not reached, the study team considered if these items should still be included. The study team had a consensus meeting and decided that if patients had consistently scored highly on an item and it had reached consensus with patients (despite it not reaching consensus overall) that it should be taken though to the next stage.
3. Median and interquartile range.
4. Evaluate the median difference between two paired samples using Wilcoxon's signed‐rank test.

The qualitative data from the free‐text questions in the rounds were analysed using a thematic content analysis approach. Free‐text responses were exported from REDCap into NVivo software (version 12) to analyse. Similar suggestions on the wording of questions, repetitive items, or reasons for numerical score were grouped together and a summary made.

### 2.6 Ethics

The study was approved by Greater Manchester West Research Ethics Committee (23/NW/0198), and informed consent was obtained for the Delphi from each participant completing the survey.

## 3 Results

### 3.1 Participants

**FIGURE 2:**
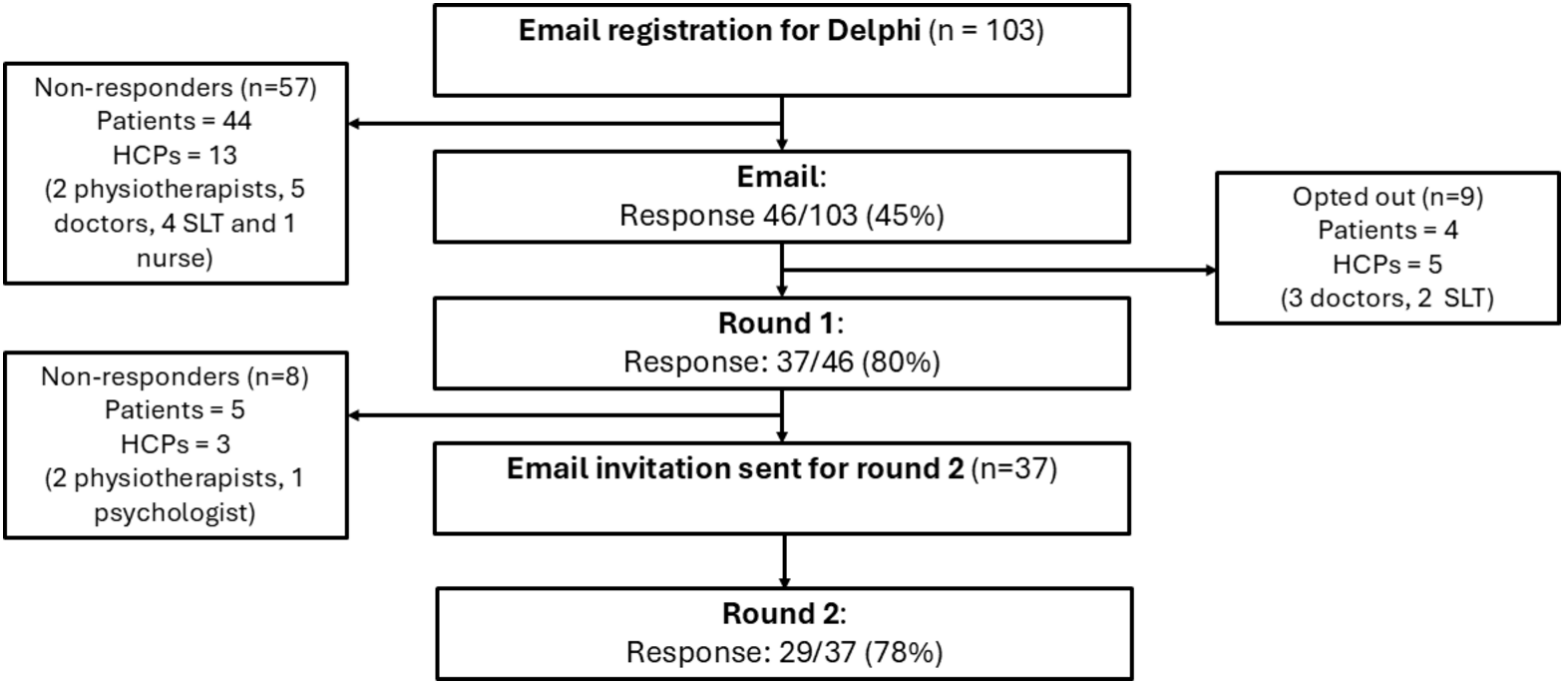
Flowchart showing responses to Delphi survey, rounds 1 and 2. HCP: Health Care Professional; SLT: Speech and language therapist.

Forty‐six participants registered for the Delphi survey of which 29 completed both rounds. Thirty‐seven participated in round 1 (23 patients and 14 health‐care professionals) and 29 participated in round 2 (18 patients and 11 health‐care professionals) as shown in Figure 2. The participants included people living with ILO (63%), respiratory physicians (10%), speech and language therapists (16%), physiotherapists (5%), a clinical psychologist (3%), and a nurse (3%). The demographics of the participants are shown in Table 1. All health‐care professionals had more than 10 years' experience working with ILO. Most participant responses were from the UK, with one health care professional responding from Australia. In round one, 30 participants were female [19/23 patients (83%) and 11/14 HCPs (79%)] and in round two, 23 participants were female [15/29 patients (83%) and 8/11 HCPs (73%)]. The mean age (SD) in round one for patients was 44 [13] years and HCPs was 40 [7] years and in round two, for patients 45 [12] years and HCPs 40 [8] years.

**TABLE 1:** Demographics.

	Round 1, *n* (%)		Round 2, *n* (%)	
All participants	37		29	
Patients and health care professionals (HCPs)	Patients	HCPs	Patients	HCPs
	23 (62)	14 (38)	18 (62)	11(38)
Male	4 (17)	3 (21)	3 (17)	3 (27)
Female	19 (83)	11 (79)	15 (83)	8 (73)
18–24 years	1 (4)			
25–34 years	3 (13)	3 (21)	3 (17)	3 (28)
35–44 years	9 (40)	7 (50)	6 (32)	4 (36)
45–54 years	5 (22)	4 (29)	4 (22)	4 (36)
55–64 years	3 (13)		3 (17)	
65–74 years	1 (4)		1 (6)	
75–84 years	1 (4)		1 (6)	
Health care professionals (HCPs)	14 (38)		11 (38)	
Respiratory Physician	4 (29)		4 (36)	
Speech and Language Therapist	6 (43)		6 (55)	
Physiotherapist	2 (14)		0	
Psychologist	1 (7)		0	
Clinical Nurse Specialist	1 (7)		1 (9)	
Patient comorbidities	23 (62)		18 (62)	
Asthma	16 (69)		13 (72)	
Chronic obstructive pulmonary disease (COPD)	1 (4)		1 (6)	
Breathing pattern disorder (BPD)	6 (26)		5 (28)	
Nasal disease	5 (22)		4 (22)	
Reflux	9 (39)		6 (33)	
Bronchiectasis	1 (4)		1 (6)	
Anxiety	7 (30)		6 (33)	
Depression	7 (30)		6 (33)	

### 3.2 Consensus and Stability Evolution

#### 3.2.1 Importance

Consensus in 70% of participants overall was reached on 30 items (37%) across the two rounds, and therefore for inclusion. Of these 30 items, over 70% consensus in both groups was reached on 20 items (25%), 7 items (9%) from the patients [’work impact’, ’exhaustion’, ’debilitated’, ’holding back’, ’held back’, ’missing out’, ‘longer term outcomes’] and 3 items (4%) from the health care professionals ['self‐conscious’, 'scared’, ’emotional impact’]. Of the items achieving consensus, 11 were reached in round one, with a further 19 in round two. The rank order achieved by each item and those achieving consensus by the end of round two are shown in Table 2. We decided that as this is a patient outcome measure that the seven items where consensus was reached by the patients only should also be taken through to be considered for inclusion; therefore, 37 items are currently included in the first draft of the ILO‐PROM questionnaire (available in the Supporting Information).

**TABLE 2:** Items rank position after completion of Round 2.

Rank	Item	% of participants scoring ‘critical’
=1	Triggers	100
=1	Sudden attacks	100
=3	Importance of diagnosis	97
=3	Avoidance	97
=5	Unpredictable	93
=5	Panic	93
=5	Adjustment	93
=8	Limitations	90
=8	Hospitalisation	90
=8	Awareness	90
=11	Anticipation	86
=11	Fear	86
=13	Work impact	83
=13	Others' reaction	83
=13	Keeping control	83
=13	Frustration	83
=17	Social impact	79
=17	Exercise impact	79
=19	Thoughts about the disease	76
=19	Missing out	76
=19	Longer term outcomes	76
=19	Holding back	76
=19	Held back	76
=19	Exhaustion	76
=19	Emotional impact	76
=19	Management	76
=27	Self‐conscious	72
=27	Scared	72
=27	Debilitated	72
=27	Believed	72

*Note:* The = sign next to the rank shows that those items are of equal rank.

#### 3.2.2 Agreement

**FIGURE 3:**
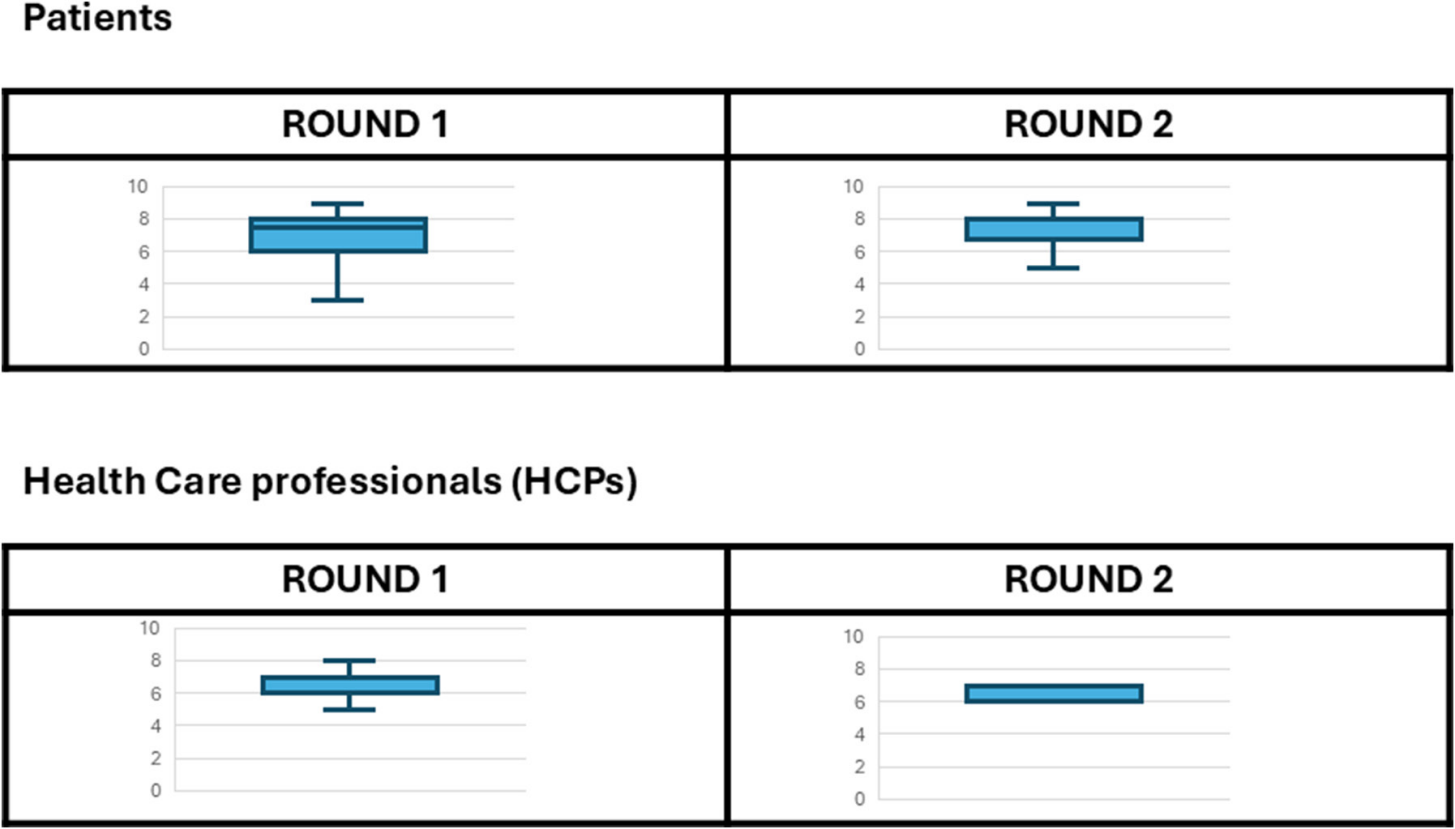
Box and whisker plot demonstrating the average change in median and interquartile range for patients and health‐care professionals between Round 1 and Round 2.

The level of within‐participant agreement was investigated between the rounds of the survey. The interquartile range reduced on most questions from round 1 to round 2 for both patients and HCPs. The average median score and interquartile range for both patients and HCPs are shown in the box and whisker plot in Figure 3. Box and whisker plots for all questions can be found in the Supporting Information. There was no statistically significant change to median scores between round 1 and round 2 for patients (Wilcoxon Signed Rank test *p* = 0.068) or HCPs (Wilcoxon Signed Rank test *p* = 0.857).

### 3.3 Qualitative Data

From the free text data, five main themes were mentioned by HCPs and patients living with ILO: (1) agreement with the question and reasons why; (2) disagreement with the question and reasons why; (3) feeling that the item had already been covered in another question; (4) feeling that the question was ambiguous; and (5) feeling that the item did not need to be captured within a questionnaire.

1. Agreement with question

Participants discussed the need for the item to be included in the PROM and their own experiences to support individual items. In response to question 1 [I worry of others' reaction to my breathing problem], patient 036B stated ‘I strongly have this anxiety every time about my ILO/stridor, people staring at me and moving away thinking I have got something that they can catch’ and HCP 044BB stated ‘In previous focus groups, the impact of other's reactions was a major theme for patients’. Several participants stated they felt the item was important to include to allow further questioning and conversation. In response to question 19 [I have negative thoughts about my breathing], HCP 051BB stated ‘opens further discussion about mental health that may need to be addressed’ and question 37 [I am scared of my breathing problem], HCP 055BB states ‘Important as this is the commonest discussion I have’. Several participants discussed how individual items support aspects of the WHO‐ICF framework with HCP 051BB stating ‘highlights social struggles’ regarding question 105 [My breathing problem stops me from attending school/college] and patient 06B stating ‘I think this is important for overall wellbeing questions’ regarding question 85 [I feel left behind in life]. Some participants demonstrated a change in how they felt about an item following an intervention, supporting its use within an outcome measure. In regard to question 83 [I feel frustrated], patient 07B stated ‘not any longer, someone has finally accepted, agreed and explained it all to me…at last’ and HCP 045BB reported ‘great to measure progress post therapy’ in regard to question 151 [I won't let my breathing problem stop me from doing things].

2 Disagreement with question

Several participants disagreed with certain questions and gave justification why. Patient 027B reported ‘I don't think you can play down this condition. There is no way of hiding an attack due to the panic and noise of an episode’ in response to question 47 [I play down my symptoms], and HCP 044BB stated ‘Our patients report the opposite of this, a tendency to try and downplay illness and protect others from worry’ in response to question 21 [I take my frustration out on others]. Some participants felt certain items were not applicable to them, such as patient 019B reporting ‘I don't attend school or college as I'm older’ in response to question 105 [my breathing stops me from attending school/college], or that the item should focus on the individual response, such as HCP 054BB stating ‘not useful question, need to concentrate on patient’ in response to question 159 [Others feel responsibility for my breathing disorder]. Others felt that the item did not add anything, and therefore gave justification for rating low importance, such as HCP 054BB stating ‘this does not add anything in my opinion to treating ILO‐ just highlights potential conflict between patients and healthcare workers…’ in response to question 11 [I have waited a long time for diagnosis].

3 Item covered in another question

Participants often felt that some of the questions were repetitive and only one was required, such as HCP 049BB stating ‘I like all these questions re: experience of feeling dismissed/ignored/frustrated etc. … but maybe not all of them…’ In response to question 109 [I feel dismissed when talking about my symptoms]. Some felt that the question was too specific and needed to be broader to cover more patients, such as HCP 040BB stating ‘this is specific and may not apply to all, my ILO/breathing problem makes me irritable may be more general’ in response to question 21 [I take my frustration out on others].

4 Ambiguous question

Participants commented on the wording of questions, feeling that the meaning was unclear at times, such as patient 027B stating ‘I was unsure of the question. If suffocated during an episode yes but if regarding mental health, I believe there should be a question to monitor mental health…’ in response to Q23 [I feel suffocated by my breathing problem]. Sometimes participants disagreed with the wording used, such as patient 029B stating ‘I wouldn't say silenced I would say, my breathing problems makes it impossible to speak during an attack’ in response to question 29 [I am silenced by my breathing problem], or some commented directly on the language used and felt it may not be understood, such as HCP 045BB stating ‘I think the concept is important but many people may not be familiar with this word’ in response to question 111 [I feel despondent].

5 Item not needing to be captured in a questionnaire

Some participants felt that certain items did not need to be discussed in a questionnaire such as HCP 054BB stating ‘not sure about antagonism to health care professionals in a PROM is needed – already captured in phrases about not being believed or listed to’ in response to question 127 [I have no belief in health care professionals] or that some questions were obvious and unlikely to change over time therefore may not be worth asking, such as HCP 047BB stating ‘Interesting to know but wonder of change could be measured or if it's just a fact’ in response to question 11 [I have waited a long time for diagnosis].

## 4 Discussion

Through a systematic process, this Delphi study identified a consensus amongst patients living with ILO and HCPs working with ILO on the items that are critical to include in the ILO‐PROM. Consensus was achieved on 30 items (by patients and HCPs) with another 7 items taken to the next round as patients felt strongly that they should be included. The 37 items achieving consensus can be themed into seven categories: symptoms, for example, ‘I feel suffocated by my breathing problem’, functioning, for example, ‘I panic when I struggle to breath’, activity limitations, for example, ‘My breathing stops me from working’, participation restriction, for example, ‘I stop myself from doing certain things’, environmental factors, for example, ‘My breathing is triggered by everyday things’ and personal factors, for example, ‘I wish I could forget about my breathing problem’ which all support the WHO‐ICF framework.

Of the 30 items that reached consensus, several were reached by both patients and HCPs (20 items), some were achieved by patients only (7 items) and a small number by HCPs only (3 items). The other 7 items which have been taken through to the next round were felt ‘critical’ to include by patients despite HCPs not changing their scores. Most items reached consensus in round 2 of the survey (19 items). Interestingly, despite HCPs being able to see the median and interquartile range of the patient scores of round 1 of the Delphi, when rating round 2, they didn't always make changes to their scores.

The median score between round 1 and round 2 for patients and HCPs mostly stayed the same and no statistically significant change was found. The interquartile range did vary between round 1 and round 2 for both patients and HCPs, with a reduced range in round 2.

The response rate to the initial email inviting participants to participate in the survey was only 46%, but the response rate to the survey itself was good (80%), with a small dropout rate in the second round (78%). A dropout rate of any size carries the risk of non‐responder bias [18]. Those who took the decision not to continue participating in the process may have had different views to those completing both rounds. Various steps were taken within the method to minimise attrition. Rounds were open for 2 weeks and reminder emails were sent after 1 week, 48 h before closing and 24 h before closing. If participants had only completed some of round 1, we made the decision to include these results and invite these participants to round 2. Only participants who completed round 1 and round 2 were included in the analysis of median and interquartile range.

Despite completing this Delphi survey, the questionnaire remains lengthy (37 items) and further steps will be required to reduce items further. Within the qualitative data analysis, several themes arose. One of the themes was that participants felt that an item had been included in another question. Combining items (if participants agree) could be a way of reducing the items. The themes will be discussed in the next stage of the study in further patient interviews and focus groups with HCPs. The next study will use the ‘think aloud’ methodology where participants will verbalise their thoughts, feelings and understanding about each of the items. Discussions will be facilitated of the themes that have come out of the Delphi study and if participants agree with these and if changes should be made. Also, further psychometric testing is required to validate the PROM including Rasch analysis to measure item responses and possible item reduction.

As far as we are aware this is the first ILO PROM that has involved patients and HCPs in each stage of its development. Using patients and clinicians in development of surveys is deemed a key part of creating a high‐quality instrument [19]. There are some limitations to this study. Patients were recruited from a single centre in the UK. Furthermore, despite attempting to get an international group of HCPs, most of these were from the UK (with only one participant from Australia). We attempted to include a range of HCPs who worked with ILO in the Delphi but had limited response from physiotherapists and psychologists, with no responses in round 2. However, speech and language therapists and respiratory physicians are predominantly responsible for the diagnosis and management of ILO, thus having the greatest responses to the Delphi, and we believe providing robust information required for this study. Using a web‐based survey meant that some patients may not have been able to participate if they did not have the technology.

The ILO‐PROM differs from current ILO PROMs in various ways. The VCDQ did not include patients and HCPs at each stage of its development. It is mostly symptom and function based and fails to include environmental and personal factors. The EILOBI included patients and HCPs in its development and covers the dimensions of function, disability and health but is for patients with exercise as a trigger and is irrelevant for those without the exercise trigger. The DI included patients and HCPs in each stage of development and includes dimensions of function, disability and health but is not specific to ILO and the questions are related to all ‘breathing problems’ not just those associated with the upper airway and throat. The ILO‐PROM provides an index that is specific to ILO, includes patients and HCPs in each stage of its development and covers all dimensions of function, disability and health in line with the WHO‐ICF framework.

In conclusion, this Delphi study identified key items to be included in a new PROM as highlighted by patients living with ILO and HCPs working with ILO. The next stage is to reduce the number of items further, through additional patient interviews, focus groups with HCPs and psychometric testing (including testing of reliability, validity, acceptability and response to change). The ILO‐PROM can then hopefully become a valuable clinical and research outcome measure in the future and be considered as one measure of a core outcome set for ILO.

## Author Contributions

**Siobhan Ludlow:** methodology, conceptualization, writing – original draft, writing – review and editing, project administration, formal analysis, funding acquisition. **Stephen Fowler:** writing – review and editing, supervision, formal analysis. **Jamie Kirkham:** methodology, writing – review and editing, supervision. **Lucie Byrne‐Davis:** writing – original draft, writing – review and editing, supervision.

## Funding

S.J.F. is supported by the NIHR Manchester Biomedical Research Centre (BRC) (grant no. NIHR203308).

## Ethics Statement

This study has received ethical clearance from the HRA and Health and Care Research Wales (HRCW) REC reference: 23/NW/0198, and informed consent was obtained for the Delphi from each participant completing the survey.

## Conflicts of Interest

The authors declare no conflicts of interest.

## Supporting information


**Appendix S1:** Inducible laryngeal obstruction patient reported Outcome measure (ILO‐PROM) Questionnaire.
**Appendix S2:** Two round Delphi survey patients.
**Appendix S3:** Two round Delphi survey health care professional's (HCPs).

## Data Availability

The data that support the findings of this study are available from the corresponding author upon reasonable request.

## References

[1] T. Halvorsen , E. S. Walstead , C. Bucca , et al., “Inducible Laryngeal Obstruction: An Official Joint European Respiratory Society and European Laryngological Society Statement,” European Respiratory Journal 50 (2017): 1602221. 28889105 10.1183/13993003.02221-2016

[2] J. Hull , V. Backer , P. Gibson , and S. Fowler , “Laryngeal Dysfunction: Assessment and Management of the Clinician,” American Journal of Respiratory and Critical Care Medicine 194, no. 9 (2016): 1062–1072. 27575803 10.1164/rccm.201606-1249CI

[3] S. Fowler , A. Thurston , B. Chesworth , et al., “The VCDQ – A Questionnaire for Symptom Monitoring in Vocal Cord Dysfunction,” Clinical & Experimental Allergy 45, no. 9 (2015): 1406–1411. 25867098 10.1111/cea.12550

[4] J. Haines , S. H. K. Chua , J. Smith , C. Slinger , A. J. Simpson , and S. J. Fowler , “Triggers of Breathlessness in Inducible Laryngeal Obstruction and Asthma,” Clinical and Experimental Allergy 50, no. 11 (2020): 1230–1237. 32713022 10.1111/cea.13715

[5] A. M. Marcinow , J. Thompson , L. A. Forrest , and B. W. DeSilva , “Irritant‐Induced Paradoxical Vocal Fold Motion Disorder: Diagnosis and Management,” Otolaryngology and Head and Neck Surgery 153, no. 6 (2015): 996–1000. 10.1177/0194599815600144 26307573

[6] S. Ludlow , L. Byrne‐Davis , and S. J. Fowler , “The Impact of Living With Inducible Laryngeal Obstruction,” Clinical and Experimental Allergy 0 (2025): 1–3. 10.1111/cea.70026 PMC12433736 40040442

[7] K. M. McConville and S. L. Thibeualt , “Patient Perceptions of the Impact of Inducible or Laryngeal Obstruction on Quality of Life,” PLoS One 19, no. 7 (2024): 1–11. 10.1371/journal.pone.0307002 PMC11251631 39012891

[8] L. Bassil , N. Pargeter , J. Fellows , and R. Howard , “Quality of Life in Inducible Laryngeal Obstruction; a Questionnaire Pilot,” Thorax 73, no. 4 (2018): A261–A262.

[9] N. Bellamy , “Principles of Clinical Outcome Assessment,” in Rheumtology, 5th ed. (Elsevier Mosby, 2011), 11–21.

[10] J. T. Olin , M. Shaffer , E. Nauman , et al., “Development and Validation of the Exercise‐Induced Laryngeal Obstruction Dyspnea‐Index (EILODI),” Journal of Allergy and Clinical Immunology 149, no. 4 (2022): 1437–1444. 34619181 10.1016/j.jaci.2021.09.027

[11] J. Gartner‐Schmidt , A. C. Shembel , T. G. Zullo , and C. A. Rosen , “Development and Validation of the Dyspnea Index (DI): A Severity Index for Upper Airway‐Related Dyspnea,” Journal of Voice 28, no. 6 (2014): 775–782. 25311596 10.1016/j.jvoice.2013.12.017

[12] S. Ludlow , L. J. Holmes , L. Simpson , et al., “A Scoping Review to Map Health Outcomes in Individuals With Inducible Laryngeal Obstruction,” International Journal of Language and Communication Disorders 61, no. 1 (2025): 1–44. 10.1111/1460-6984.70169 PMC12712271 41408678

[13] World Health Organisation , International Classification of Functioning, Disability, and Health (ICF) (WHO, 2001), https://www.who.int/standards/classifications/international‐classification‐of‐functioning‐disability‐and‐health.

[14] S. Keeney , F. Hasson , and H. McKenna , The Delphi Technique in Nursing and Health Research (Wiley‐Blackwell, 2011), 10.1002/9781444392029.

[15] S. Junger , S. A. Payne , J. Brine , et al., “Guidance on Conducting and Reporting Delphi Studies (CREDES) in Palliative Care: Recommendations Based on a Methodological Systematic Review,” Palliative Medicine 31, no. 8 (2017): 684–706. 28190381 10.1177/0269216317690685

[16] P. A. Harris , R. Taylor , R. Thielke , and J. Payne , “Research Electronic Data Capture (REDCap) – A Metadata‐Driven Methodology and Workflow Process for Providing Translational Research Informatics Support,” Journal of Biomedical Informatics 42, no. 2 (2009): 377–381. 18929686 10.1016/j.jbi.2008.08.010 PMC2700030

[17] E. A. Holey , J. L. Feeley , J. Dixon , and V. J. Whittaker , “An Exploration of the Use if Simple Statistics to Measure Consensus and Stability in Delphi Studies,” BMC Medical Research Methodology 29, no. 7 (2007): 52. 10.1186/1471-2288-7-52 PMC2216026 18045508

[18] J. Greatorex and T. Dexter , “An Accessible Analytical Approach for Investigating What Happens Between the Rounds of a Delphi Study,” Journal of Advanced Nursing 32, no. 4 (2000): 1016–1024. 11095243

[19] J. Khada , C. McAlinden , and K. Pesudovs , “Quality Assessment of Ophthalmic Questionnaires: Review and Recommendations,” Optometry and Vision Science 90, no. 8 (2013): 720–744. 23873034 10.1097/OPX.0000000000000001

