# Supplementary material for: Preliminary Development of a Patient‐Reported Outcome Measure for Inducible Laryngeal Obstruction: Findings From a Delphi Process
Source: Respirology. 2026 May 19;31(8):840–7. doi: 10.1002/resp.70262 (PMC13432505; doi:10.1002/resp.70262)
Supplement: Supplementary file 1 — Appendix S1: Inducible laryngeal obstruction patient reported Outcome measure (ILO‐PROM) Questionnaire. Appendix S2: Two round Delphi survey patients. Appendix S3: Two round Delphi survey health care professional's (HCPs). [file RESP-31-840-s001.docx]

**Supporting information**

**Appendix S1:** Inducible Laryngeal Obstruction Patient Reported Outcome Measure (ILO-PROM) Questionnaire

**Appendix S2:** Two Round Delphi Survey Patients

**Appendix S3:** Two Round Delphi Survey Health Care Professional’s (HCPs)

|  | **1** | **2** | **3** | **4** | **5** | **6** | **7** |
| --- | --- | --- | --- | --- | --- | --- | --- |
|  | **Strongly disagree** | **Disagree** | **Somewhat disagree** | **Neither agree nor disagree** | **Somewhat agree** | **Agree** | **Strongly agree** |
| 1. **My breathing stops me from working** |  |  |  |  |  |  |  |
| 1. **My breathing problem can be unpredictable** |  |  |  |  |  |  |  |
| 1. **My breathing problem is triggered by everyday things** |  |  |  |  |  |  |  |
| 1. **I have negative thoughts about my breathing problem** |  |  |  |  |  |  |  |
| 1. **My breathing problem causes sudden ‘’attacks’’** |  |  |  |  |  |  |  |
| 1. **I tend to stay at home because of my breathing problem** |  |  |  |  |  |  |  |
| 1. **I am self-conscious** |  |  |  |  |  |  |  |
| 1. **I am scared of my breathing problem** |  |  |  |  |  |  |  |
| 1. **I panic when I struggle to breathe** |  |  |  |  |  |  |  |
| 1. **I fear other’s reaction to my breathing problem** |  |  |  |  |  |  |  |
| 1. **I feel I am missing out on things because of my breathing problem** |  |  |  |  |  |  |  |
| 1. **I am unsure of the long-term outcome of my breathing problem** |  |  |  |  |  |  |  |
| 1. **I am limited by my breathing problem** |  |  |  |  |  |  |  |
| 1. **I struggle to keep control of my breathing** |  |  |  |  |  |  |  |
| 1. **Getting a diagnosis is important** |  |  |  |  |  |  |  |
| 1. **I have had to attend hospital because of my breathing problem** |  |  |  |  |  |  |  |
| 1. **I stop myself from doing certain things** |  |  |  |  |  |  |  |
| 1. **I feel held back by my breathing problem** |  |  |  |  |  |  |  |
| 1. **I feel frustrated** |  |  |  |  |  |  |  |
| 1. **I am fearful of my breathing problem** |  |  |  |  |  |  |  |
| 1. **I feel exhausted** |  |  |  |  |  |  |  |
| 1. **I am unable to exercise** |  |  |  |  |  |  |  |
| 1. **I feel low in mood because of my breathing problem** |  |  |  |  |  |  |  |
| 1. **I feel debilitated by my breathing problem** |  |  |  |  |  |  |  |
| 1. **I do not feel believed** |  |  |  |  |  |  |  |
| 1. **I avoid certain triggers** |  |  |  |  |  |  |  |
| 1. **I anticipate the breathing attacks happening** |  |  |  |  |  |  |  |
| 1. **I have had to adjust my lifestyle** |  |  |  |  |  |  |  |
| 1. **I am aware of my triggers** |  |  |  |  |  |  |  |
| 1. **I have learnt how to manage my breathing problem** |  |  |  |  |  |  |  |
| 1. **I feel withdrawn** |  |  |  |  |  |  |  |
| 1. **I wish I could forget about my breathing problem** |  |  |  |  |  |  |  |
| 1. **I want to be normal again** |  |  |  |  |  |  |  |
| 1. **I feel suffocated by my breathing problem** |  |  |  |  |  |  |  |
| 1. **I often cancel events (social/work)** |  |  |  |  |  |  |  |
| 1. **I am disappointed this is something I must live with** |  |  |  |  |  |  |  |
| 1. **I struggle to communicate my needs to others** |  |  |  |  |  |  |  |

**Appendix S1: Inducible Laryngeal Obstruction Patient Reported Outcome Measure (ILO-PROM) Questionnaire**

**Appendix S2: Two Round Delphi Survey Patients**

|  | **ROUND 1** | **ROUND 2** |
| --- | --- | --- |
| **Q1** | 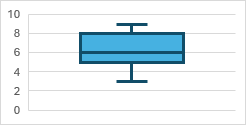 | 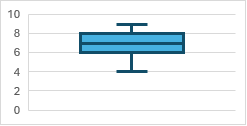 |
| **Q3** | 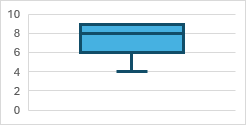 | 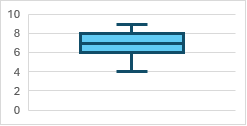 |
| **Q5** | 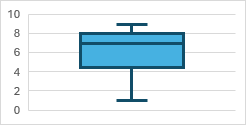 | 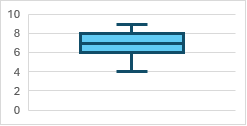 |
| **Q7** | 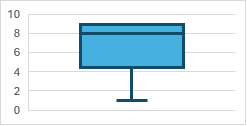 | 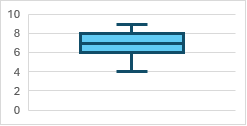 |
| **Q9** | 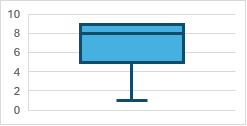 | 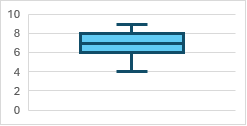 |
| **Q11** | 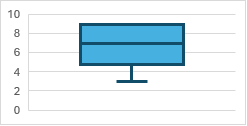 | 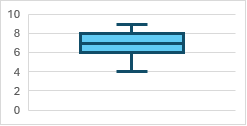 |
| **Q13** | 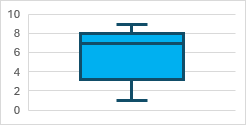 | 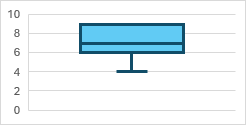 |
| **Q15** | 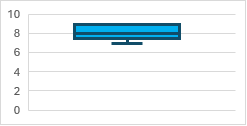 | 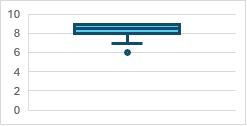 |
| **Q17** | 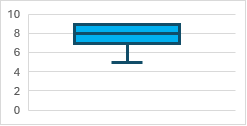 | 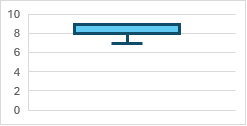 |
| **Q19** | 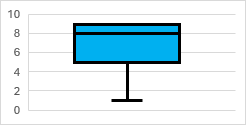 | 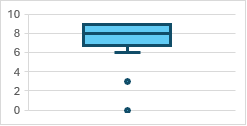 |
| **Q21** | 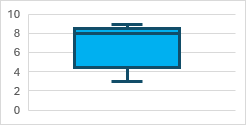 | 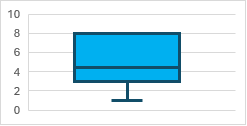 |
| **Q23** | 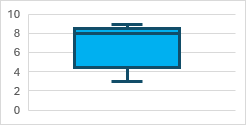 | 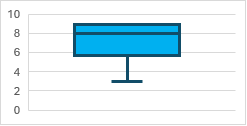 |
| **Q25** | 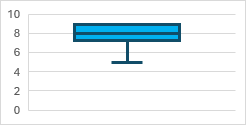 | 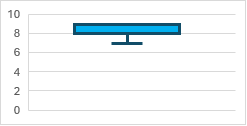 |
| **Q27** | 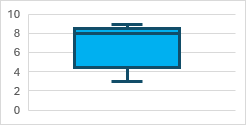 | 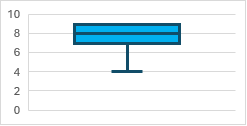 |
| **Q29** | 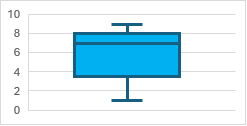 | 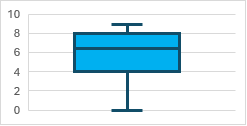 |
| **Q31** | 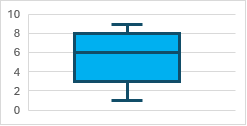 | 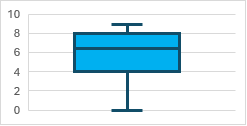 |
| **Q33** | 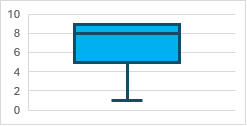 | 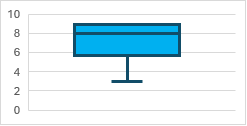 |
| **Q35** | 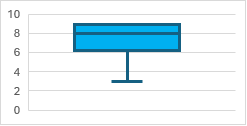 | 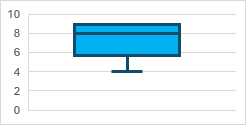 |
| **Q37** | 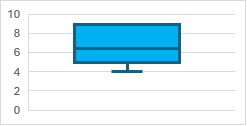 | 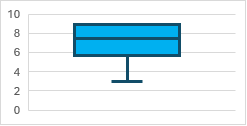 |
| **Q39** | 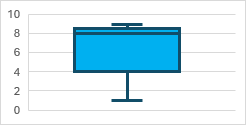 | 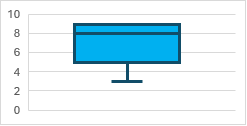 |
| **Q41** | 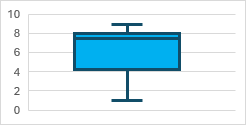 | 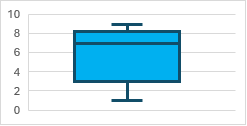 |
| **Q43** | 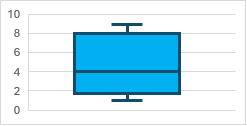 | 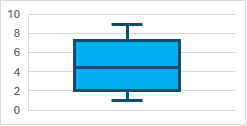 |
| **Q45** | 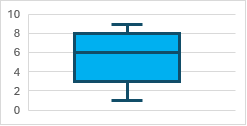 | 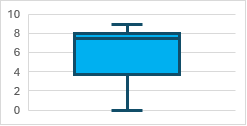 |
| **Q47** | 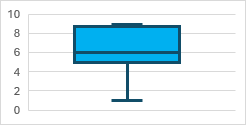 | 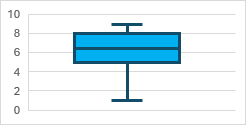 |
| **Q49** | 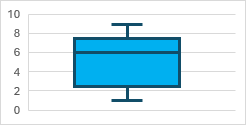 | 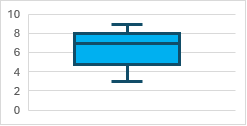 |
| **Q51** | 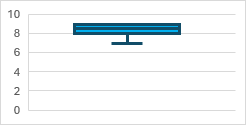 | 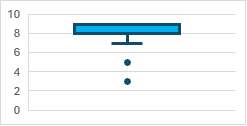 |
| **Q53** | 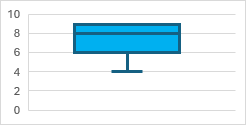 | 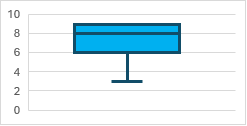 |
| **Q55** | 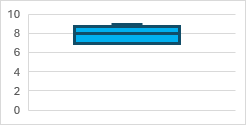 | 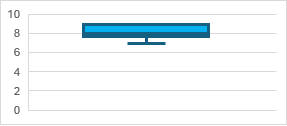 |
| **Q57** | 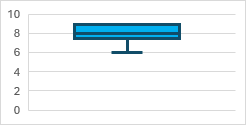 | 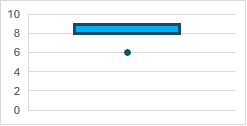 |
| **Q59** | 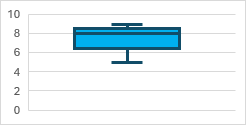 | 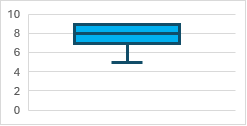 |
| **Q61** | 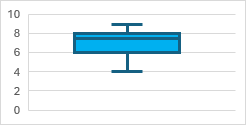 | 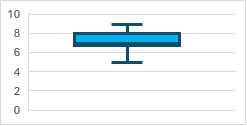 |
| **Q63** | 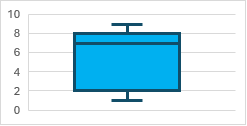 | 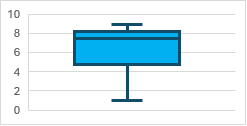 |
| **Q65** | 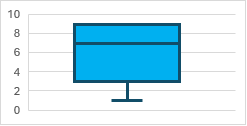 | 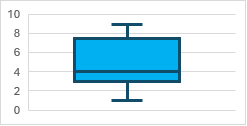 |
| **Q67** | 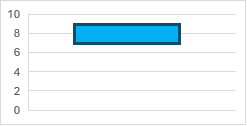 | 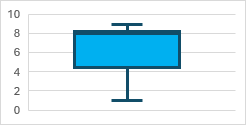 |
| **Q69** | 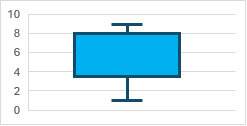 | 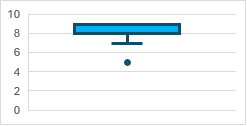 |
| **Q71** | 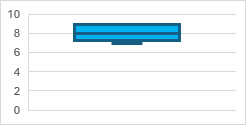 | 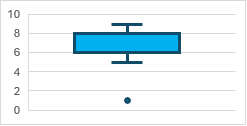 |
| **Q73** | 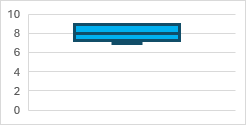 | 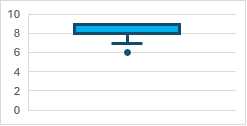 |
| **Q75** | 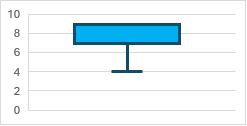 | 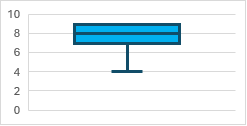 |
| **Q77** | 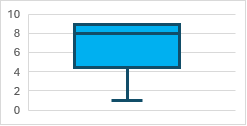 | 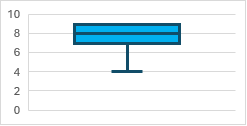 |
| **Q79** | 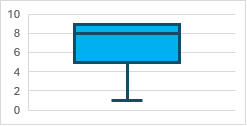 | 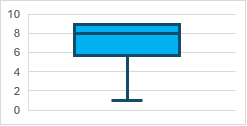 |
| **Q81** | 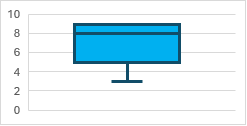 | 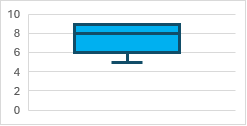 |
| **Q83** | 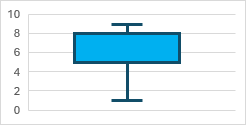 | 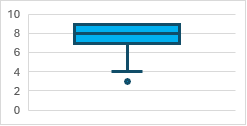 |
| **Q85** | 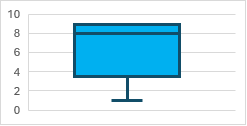 | 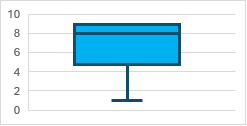 |
| **Q87** | 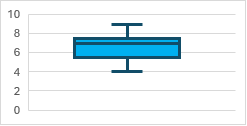 | 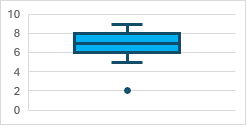 |
| **Q89** | 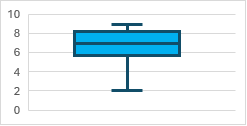 | 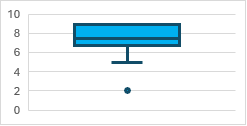 |
| **Q91** | 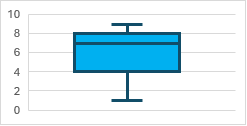 | 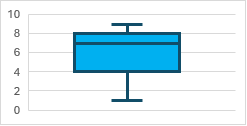 |
| **Q93** | 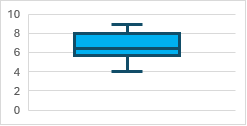 | 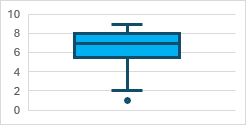 |
| **Q95** | 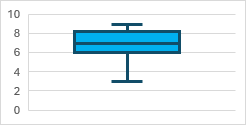 | 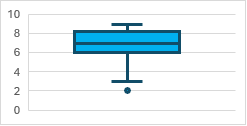 |
| **Q97** | 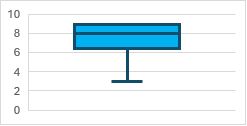 | 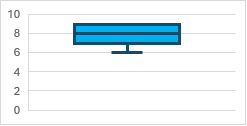 |
| **Q99** | 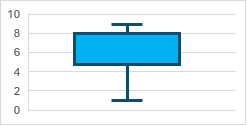 | 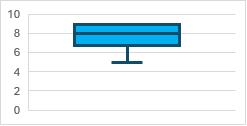 |
| **Q101** |  |  |
| **Q103** |  |  |
| **Q105** |  |  |
| **Q107** |  |  |
| **Q109** |  |  |
| **Q111** |  |  |
| **Q113** |  |  |
| **Q115** |  |  |
| **Q117** |  |  |
| **Q119** |  |  |
| **Q121** |  |  |
| **Q123** |  |  |
| **Q125** |  |  |
| **Q127** |  |  |
| **Q129** |  |  |
| **Q131** |  |  |
| **Q133** |  |  |
| **Q135** |  |  |
| **Q137** |  |  |
| **Q139** |  |  |
| **Q141** |  |  |
| **Q143** |  |  |
| **Q145** |  |  |
| **Q147** |  |  |
| **Q149** |  |  |
| **Q151** |  |  |
| **Q153** |  |  |
| **Q155** |  |  |
| **Q157** |  |  |
| **Q159** |  |  |

**Appendix S3: Two Round Delphi Survey Health Care Professional’s (HCPs)**

|  | **ROUND 1** | **ROUND 2** |
| --- | --- | --- |
| Q1 |  |  |
| Q3 |  |  |
| Q5 |  |  |
| Q7 |  |  |
| Q9 |  |  |
| Q11 |  |  |
| Q13 |  |  |
| Q15 |  |  |
| Q17 |  |  |
| Q19 |  |  |
| Q21 |  |  |
| Q23 |  |  |
| Q25 |  |  |
| Q27 |  |  |
| Q29 |  |  |
| Q31 |  |  |
| Q33 |  |  |
| Q35 |  |  |
| Q37 |  |  |
| Q39 |  |  |
| Q41 |  |  |
| Q43 |  |  |
| Q45 |  |  |
| Q47 |  |  |
| Q49 |  |  |
| Q51 |  |  |
| Q53 |  |  |
| Q55 |  |  |
| Q57 |  |  |
| Q59 |  |  |
| Q61 |  |  |
| Q63 |  |  |
| Q65 |  |  |
| Q67 |  |  |
| Q69 |  |  |
| Q71 |  |  |
| Q73 |  |  |
| Q75 |  |  |
| Q77 |  |  |
| Q79 |  |  |
| Q81 |  |  |
| Q83 |  |  |
| Q85 |  |  |
| Q87 |  |  |
| Q89 |  |  |
| Q91 |  |  |
| Q93 |  |  |
| Q95 |  |  |
| Q97 |  |  |
| Q99 |  |  |
| Q101 |  |  |
| Q103 |  |  |
| Q105 |  |  |
| Q107 |  |  |
| Q109 |  |  |
| Q111 |  |  |
| Q113 |  |  |
| Q115 |  |  |
| Q117 |  |  |
| Q119 |  |  |
| Q121 |  |  |
| Q123 |  |  |
| Q125 |  |  |
| Q127 |  |  |
| Q129 |  |  |
| Q131 |  |  |
| Q133 |  |  |
| Q135 |  |  |
| Q137 |  |  |
| Q139 |  |  |
| Q141 |  |  |
| Q143 |  |  |
| Q145 |  |  |
| Q147 |  |  |
| Q149 |  |  |
| Q151 |  |  |
| Q153 |  |  |
| Q155 |  |  |
| Q157 |  |  |
| Q159 |  |  |
